# Editorial: Risk behavior and its connection to addiction

**DOI:** 10.3389/fpsyt.2023.1229912

**Published:** 2023-07-06

**Authors:** Huixi Dong, Na Zhong, Jing Zhang, Qian Xiao

**Affiliations:** ^1^Mental Health Centre, Xiangya Hospital, Central South University, Changsha, China; ^2^Shanghai Mental Health Center, Shanghai Jiao Tong University School of Medicine, Shanghai, China; ^3^Department of Computer Science, Donald Bren School of Information and Computer Sciences, University of California, Irvine, Irvine, CA, United States

**Keywords:** behavioral addiction, non-suicidal self-injury (NSS), problematic internet pornography use (PIPU), gambling disorder (GD), alcohol withdrawal syndrome (AWS), internet addiction (IA)

The scope of addictive disorders expands in recent years. Both the International Classification of Diseases (1) and the Diagnostic and Statistical Manual of Mental Disorders (2) have classified behavioral addictions into the respective substance dependence categories. Despite gambling disorder and internet gaming disorder (IGD), other repetitive, maladaptive and out-of-control behaviors, including compulsive sexual behaviors, self-injury behaviors, binge eating and compulsive buying might also be considered in the spectrum of addictions in the future. Seven papers have examined various psychological, behavioral, and imaging changes associated with several addictive disorders in this Research Topic.

Two papers discussed the influencing and related factors of non-suicidal self-injury (NSSI) in samples of adolescents. Reinhardt et al. have investigated the relationship between interpersonal and intrapersonal motivations of NSSI with several indicators and psychopathological characteristics of NSSI severity in clinical institutions. They have found intrapersonal NSSI functions were related to NSSI severity and many psychopathological features, including the comorbidity of mental disorders, mood disorder, more internalizing symptoms of mental disorders, and more self-critical rumination. Li et al. have found that hospitalized adolescents with NSSI had a higher prevalence of childhood maltreatment. Moreover, impulsivity plays a mediating role between NSSI behavior and childhood maltreatment.

In another study, Salehi et al. also evaluated impulsivity in behavioral addiction. A significant correlation between impulsivity and internet addiction was found in the medical students of Golestan University of Medical Sciences, Golestan, Iran. A significant correlation was also shown between impulsivity and internet addiction, both in males and females. Restraint index was positively correlated with internet addiction in female students.

Liu et al. (3) demonstrated that protective or risk-enhancing effects on alcohol withdrawal syndrome (AWS)-related impulsivity and aggression might depend on specific allelic combinations of ZNF804A and mTOR. Single gene analysis revealed the naturally occurring allelic variation in ZNF804A rs1344706 (A allele/CC homozygote) and mTOR rs1057079 (C allele/TT homozygote) have been intensively implicated in AWS-related impulsivity and aggression. In allelic group, MANOVA revealed a significant gene x gene interaction with results showing that risk varied systematically depending on both ZNF804A and mTOR alleles. Further results revealed a significant interactive effect of ZNF804A rs1344706 and mTOR rs7525957 on motor impulsivity and physical aggression, and the ZNF804A rs1344706 gene variant has significant effects on motor impulsivity and physical aggression only in mTOR rs7525957 TT homozygous carriers.

One study (Wang and Huang) examined the components of attentional processing of sexual stimuli by an exogenous cueing task, which aimed to distinguish between high and low propensity attention participation and disengagement in subjects with problematic internet pornography use (PIPU). Erotic and neutral images were presented using two different stimulus presentation times (100 and 500 ms) to distinguish early and late stages of attentional bias. The results showed that individuals with a higher tendency of PIPU showed increased attentional engagement to erotic stimuli during early stages of attentional processing (100 ms), followed by attentional avoidance during later stages of attentional processing (500 ms). Furthermore, the severity of PIPU symptoms was positively correlated with attentional engagement scores in the short picture-time trials, whereas it was weakly negatively correlated with attentional disengagement scores in long picture time trials.

In the last two papers, authors have performed resting-state functional magnetic resonance imaging (fMRI) in subjects with IGD and gambling disorder. Zhou et al. have first examined the effective connectivity within and between empathy and gambling networks in disordered gamblers and healthy controls (HCs). Compared with HCs group, disordered gamblers showed more excitatory and effective connectivity within the gambling network, with more excitatory effective connectivity from the empathy network to the gambling network. While the inhibitory and effective connectivity from the gambling network to the empathy network decreased. Liu et al. have investigated individuals with IGD and interviewed non-dropout subjects who met IGD (PER-IGD) but no longer met IGD (RE-IGD) after 1 year. Compared to RE-IGD individuals, the orbitofrontal cortex (OFC), the precuneus and the dorsolateral prefrontal cortex (DLPFC) in the PER-IGD individuals were reduced. In both groups, there was a significant positive correlations between mean ReHo values in the precuneus and self-reported craving scores for gaming. In addition, similar differences in brain structure and cue-craving were found between the PER-IGD and RE-IGD individuals, especially in brain regions related to reward processing and inhibitory control (including DLPFC, anterior cingulate gyrus, insula, OFC, precuneus, and superior frontal gyrus).

In conclusion, we have collected seven studies exploring the effects of psychological and environmental risk factors on risky behaviors and their underlying additive mechanisms, as well as neurobiological or neuroimaging evidence of addictive behaviors or related clinical symptoms. We hope that these studies can provide some evidence of the causes or intervention directions of behavioral addiction.

## Author contributions

HXD has contributed to the writing of this editorial. All authors contributed to the article and approved the submitted version.
